# High Pressure-Induced mtDNA Alterations in Retinal Ganglion Cells and Subsequent Apoptosis

**DOI:** 10.3389/fncel.2016.00254

**Published:** 2016-11-24

**Authors:** Sheng-Hai Zhang, Feng-Juan Gao, Zhong-Mou Sun, Ping Xu, Jun-Yi Chen, Xing-Huai Sun, Ji-Hong Wu

**Affiliations:** ^1^Eye and ENT Hospital, State Key Laboratory of Medical Neurobiology, Institutes of Brain Science, Shanghai Medical College, Fudan UniversityShanghai, China; ^2^Shanghai Key Laboratory of Visual Impairment and RestorationShanghai, China; ^3^Molecular Biology and Biochemistry Department, Wesleyan UniversityMiddletown, CT, USA; ^4^Schepens Eye Research Institute, Wesleyan UniversityMiddletown, CT, USA; ^5^Key Laboratory of Myopia, Ministry of Health, Fudan UniversityShanghai, China

**Keywords:** retinal ganglion cells, hydrostatic pressure, mitochondrial DNA, mutation, mitochondrial dysfunction

## Abstract

**Purpose**: Our previous study indicated that mitochondrial DNA (mtDNA) damage and mutations are crucial to the progressive loss of retinal ganglion cells (RGCs) in a glaucomatous rat model. In this study, we examined whether high pressure could directly cause mtDNA alterations and whether the latter could lead to mitochondrial dysfunction and RGC death.

**Methods**: Primary cultured rat RGCs were exposed to 30 mm Hg of hydrostatic pressure (HP) for 12, 24, 48, 72, 96 and 120 h. mtDNA alterations and mtDNA repair/replication enzymes OGG1, MYH and polymerase gamma (POLG) expressions were also analyzed. The RGCs were then infected with a lentiviral small hairpin RNA (shRNA) expression vector targeting POLG (POLG-shRNA), and mtDNA alterations as well as mitochondrial function, including complex I/III activities and ATP production were subsequently studied at appropriate times. Finally, RGC apoptosis and the mitochondrial-apoptosis pathway-related protein cleaved caspase-3 were detected using a Terminal deoxynucleotidyl transferase dUTP nick end-labeling (TUNEL) assay and western blotting, respectively.

**Results**: mtDNA damage was observed as early as 48 h after the exposure of RGCs to HP. At 120 h after HP, mtDNA damage and mutations significantly increased, reaching >40% and 4.8 ± 0.3-fold, respectively, compared with the control values. Twelve hours after HP, the expressions of OGG1, MYH and POLG mRNA in the RGCs were obviously increased 5.02 ± 0.6-fold (*p* < 0.01), 4.3 ± 0.2-fold (*p* < 0.05), and 0.8 ± 0.09-fold (*p* < 0.05). Western blot analysis showed that the protein levels of the three enzymes decreased at 72 and 120 h after HP (*p* < 0.05). After interference with POLG-shRNA, the mtDNA damage and mutations were significantly increased (*p* < 0.01), while complex I/III activities gradually decreased (*p* < 0.05). Corresponding decreases in membrane potential and ATP production appeared at 5 and 6 days after POLG-shRNA transfection respectively (*p* < 0.05). Increases in the apoptosis of RGCs and cleaved caspase-3 protein expression were observed after mtDNA damage and mutations.

**Conclusions**: High pressures could directly cause mtDNA alterations, leading to mitochondrial dysfunction and RGC death.

Glaucoma neurodegeneration, a leading cause of irreversible blindness worldwide, is characterized by aggressive death of retinal ganglion cells (RGCs) and visual field impairment (Gupta and Yücel, 2007; Osborne, 2008). The mechanisms involved in RGC death in glaucoma are complicated. The elevated intraocular pressure (IOP) is considered to be the major risk factor (Osborne, 2008; Ju et al., 2015; Yang et al., 2016), and lowering the IOP is thus far the only relatively effective treatment clinically (Chen et al., 2013; Itakura et al., 2015). However, lowering the IOP is not sufficient to prevent or delay progressive vision loss, and the molecular mechanisms underlying accelerated RGC loss, even when the raised IOP is lowered to a normal level for many years, remain poorly understood (Gupta and Yücel, 2007; Osborne, 2010). Further insight into glaucoma pathogenesis is critical for exploring novel therapeutic strategies for protecting RGCs and rescuing vision in glaucoma patients.

Increasing evidence has shown that mitochondria disorders play an important role in the development of glaucoma (Lee et al., 2011; McElnea et al., 2011; Lascaratos et al., 2012). It has been reported that OPA1, a protein required for mitochondrial fusion, was down-regulated in the peripheral blood of patients with primary open angle glaucoma (Abu-Amero et al., 2006; Bosley et al., 2011). The neuroprotective effects against elevated pressure exerted by lithium chloride and iNOS were involved in regulating the mitochondrial dynamic proteins DRP1 and OPA1, respectively (Dai et al., 2011). Recently, Ju et al. (2015) found that increasing mitochondrial fission, volume density and length protected astrocytes in glaucomatous neurodegeneration. Moreover, many studies have indicated that mitochondrial abnormalities occur in primary open-angle glaucoma patients, including increasing mitochondrial DNA (mtDNA) pathogenic mutations and content as well as decreasing mitochondrial respiratory activity (Abu-Amero et al., 2011; Nucci et al., 2013; Sundaresan et al., 2015). Our previous studies have shown that progressively increased mtDNA damage and mutations contributed to aggressive RGC death in rat model of experimental glaucoma, and the prevention of mtDNA alterations improved RGC survival after IOP elevation (Wu et al., 2015). However, the underlying mechanisms triggering mtDNA mutations and damage in glaucoma remain unknown because of the complexity of the *in vivo* microenvironment of the retina after IOP elevation.

The aim of this study was to determine whether elevated pressure itself, as the major risk factor for glaucoma, directly induced mtDNA alterations, which then led to subsequent mitochondrial dysfunction and RGC death. We investigated mutations of and damage to mtDNA under hydrostatic pressure (HP) using primarily cultured rat RGCs *in vitro* to eliminate interference factors (such as oxidative stress, glutamate excitotoxicity, or reperfusion injury) that existed *in vivo*, and we further explored the changes in mitochondrial function and RGC apoptosis induced by mtDNA alterations. We demonstrated that high pressure could directly damage mtDNA. Increasing mtDNA alterations by blocking mtDNA repair/replication enzymes could ultimately result in mitochondrial dysfunction and RGC apoptosis. These results further provided a novel mechanism of RGC death in glaucoma: an elevated IOP causes RGC mtDNA alterations, followed by mitochondrial dysfunction and cell apoptosis.

## Materials and Methods

### Ethics Statement

All animals (obtained from SLAC Laboratory Animal Co., Ltd. Shanghai, China) were handled and housed according to the ARVO Statement for the Use of Animals in Ophthalmic and Vision Research. All procedures followed the Declaration of Helsinki, accorded with the guidelines of Fudan University on the ethical use of animals, and all endeavors were done to minimize animal suffering. A total of 420 rats were used.

### Cell Culture and Treatment

The isolation protocol for purified RGCs followed our previously described two-step immunopanning-magnetic separation method (Wu et al., 2015; Gao et al., 2016). Briefly, 2-day-old Sprague-Dawley rats were sacrificed to obtain the retinas. The retinas were removed and dissociated in 4.5 units/mL of papain solution (#3126, Worthington, Lakewood, NJ, USA). The cell suspensions were then transferred to a petri dish coated with anti-macrophage antibody (#CLAD51240; Cedarlane Laboratories, Hornby, ON, Canada) and CD90.1 MicroBeads (Miltenyi Biotec, GmbH, Bergisch Gladbach, Germany) successively, and RGCs were then collected by an MS column (Miltenyi Biotec GmbH) in the magnetic fields of a MiniMACS (Miltenyi Biotec GmbH). The isolated RGCs were then seeded onto 6-well plates treated with poly-D-lysine (#P6407; Sigma-Aldrich, St. Louis, MO, USA) and mouse-laminin (#3400-010-01; Trevigen Inc., Gaithersburg, MD, USA). The cultures were maintained at 37°C in a humidified environment containing 10% CO_2_. To mimic chronically elevated IOP, the cells were exposed to 30 mm Hg of HP using a closed, pressurized chamber equipped with a manometer for 12, 24, 48, 72 and 120 h, as we previously reported (Wu et al., 2013). Subsequently, the cells were collected for further analysis.

To induce mtDNA mutations and damage *in vitro*, the cultured RGCs were transfected with lentivirus-small harpin polymerase gamma (shPOLG; Santa Cruz Biotechnology, CA, USA). Lentivirus-scrambled RNA was used as a control (Lentivirus-SC RNA, Santa Cruz Biotechnology). As previously reported (Tewari et al., 2012), the cells were incubated with the lentivirus-shPOLG or SC RNA for 4 days, washed with phosphate-buffered saline (PBS, Gibco, Grand Island, NY, USA), and incubated for 6 additional days. The transfection efficiency was evaluated by quantifying POLG gene expression. mtDNA was examined using Long-extension (LX)-PCR and mtDNA mutation assays. Mitochondrial function was assessed by examining complex activity, ATP production and mitochondrial membrane potential (Δψm).

### Mitochondria Isolation

The cultured RGCs were harvested and mitochondria were isolated using Mitochondria Isolation Kit (Pierce Biotechnology, Rockford, IL, USA) as previously reported (Wu et al., 2015). The isolated mitochondrial pellet was resuspended in 100 μL of mannitol (210 mM), sucrose (70 mM), HEPES (5 mM), EGTA (1 mM) and fatty-acid-free bovine serum albumin (BSA, 0.5% (w/v) Sigma-Aldrich, St. Louis, MO, USA) solution.

### Western Blot Analysis

RGC protein extractions and Western blot analysis were performed as previously reported (Wu et al., 2015). Briefly, cultured RGCs were lysed and total proteins were extracted on ice by use of cell lysis buffer (Cell Signaling Technology, Boston, MA, USA) and protease inhibitor cocktail (Sigma-Aldrich, St. Louis, MO, USA). The protein concentration was quantified using a BCA protein assay kit (Thermo Fisher Scientific, Rockford, IL, USA), and then the proteins were separated by SDS-polyacrylamide gel electrophoresis and the separated proteins were electrotransferred to polyvinylidene difluoride membranes. After blocking with 5% non-fat milk for 20 min, the membranes were incubated overnight at 4°C with primary antibodies against DNA POLG (Abcam, Cambridge, MA, USA) and cleaved caspase-3 antibody (Abcam, Cambridge, MA, USA). The loading controls included β-actin (Abcam, Cambridge, MA, USA) for the total proteins and Cox IV (Molecular Probes, Rockford, IL, USA) for mitochondria. Secondary antibodies included HRP-conjugated goat anti-rabbit antibody (Millipore, MA, USA) and HRP-conjugated goat anti-mouse antibody (Millipore, MA, USA). The resulting western blots were exposed to film (Hyperfilm ECL, Thermo Fisher Scientific, Rockford, IL, USA) and were analyzed using the Kodak Imaging System (Kodak 440CF). The intensity of the band was quantified by densitometry using ImageJ software (NIH, Bethesda, MD, USA).

### DNA Isolation

The total DNA and mtDNA were extracted from the RGCs as described in our previously reported (Wu et al., 2015). Shortly, the total DNA was isolated using the DNeasy blood and tissue kit (QIAGEN, Duess eldorf, Germany). mtDNA were obtained from the isolated mitochondria by use of mitochondrial lysis buffer containing proteinase K (0.2 mg/ml), SDS (0.5%), Tris-HCl (10 mM) and 0.05 M EDTA. The amount of DNA was determined using the Quant-iT dsDNA assay kit (Invitrogen, Carlsbad, CA, USA).

### Quantification of mtDNA Mutation

mtDNA mutation frequency was detected by random mutation capture assay as described in our published article (Wu et al., 2015). In summary, two primer pairs were designed for PCR reactions. One is flanking the Taq1 sites at mtDNA positions 1427 and 8335, the other primer pair does not flank a TaqI restriction sites as controls. PCR amplification was performed in 25 μL reactions, containing 2 μL of 10 μM forward and reverse primers, 0.2 μL of uracil DNA glycosylase (New England Biolabs, Beverly, MA, USA), 12.5 μL 2× Brilliant SYBR Green I Master Mix (Stratagene, La Jolla, CA, USA) and 3.3 μL H_2_O. Then the PCR product was digested with TaqI restriction enzyme and the mtDNA mutation frequency was determined by analysis of the band size of gel electrophoresis. The mutation frequency was showed as the mutation number per million bases.

LX-PCR assay was performed to determine DNA damage using GeneAmp XL PCR kit (Applied Biosystems, CA, USA) as previously described (Wu et al., 2015). DNA damage was quantified by comparing the ratio between the long and short fragments of PCR amplicons (mtDNA = 210 bp/13.4 kb).

### Quantitative PCR Analysis

The total RNA was extracted by use of TRIzol reagent (Invitrogen, Carlsbad, CA, USA) and cDNA was generated using a SuperScript First-Strand Synthesis kit (Takara, Tokyo, Japan) according to the manufacturer’s instructions. The mRNA expressions of Polg, Ogg1 and Myh were assessed by SYBR Green-based real-time PCR using an Applied Biosystems, 7500fast (Life Technologies, Grand Island, NY, USA). The β-actin was acted as a normalized control. Amplifications were performed in duplicate well in two independent experiments, and the transcripts were quantified by the ΔΔC (t) method.

### Mitochondrial Function Assay

Mitochondrial membrane potential (Δψm) was evaluated using MitoProbe JC-1 dye (Invitrogen), according to the manufacturer’s specifications. In brief, RGCs cultured in a six-well plate with different treatments were incubated with JC-1 for 30 min and were visualized using an inverted fluorescence microscope (Leica; green: 488 nm excitation/530 nm emission; red: 550 nm excitation/590 nm emission). Quantitative analysis was performed using ImageJ software, and Δψm was indicated by the ratio of the mean red fluorescence to the mean green fluorescence. According to this assay, mitochondrial depolarization was indicated by an increase in the green-to-red fluorescence intensity ratio.

The activities of mitochondrial complex I and III were detected using previously described methods (Birch-Machin and Turnbull, 2001; Rosca et al., 2005; Yao et al., 2011; Wu et al., 2015) with a Synergy H1 plate reader (BioTek Winooski, VT, USA) using the kinetics mode of Gen5 software (BioTek). Briefly, complex I activity was determined by following the decrease in absorbance to the NADH oxidation at 340 nm. Complex III activity was measured by monitoring the cytochrome c reduction at 550 nm. Enzymatic activities are expressed as nmol/mg protein/min.

Rate of ATP production was assayed using bioluminescence assay kit (ENLITEN ATP assay system; Promega, Madison, WI, USA), followed by the manufacturer’s protocol in our lab (Wu et al., 2015). The ATP content was measured using a microplate reader (BioTek Synergy HT) and was standardized to the total proteins of mitochondria. The results were calculated from three independent ATP standards run in duplicate.

### Terminal Deoxynucleotidyl Transferase dUTP Nick End-Labeling (TUNEL) Assay

A terminal deoxynucleotidyl transferase-mediated dUTP nick end-labeling (TUNEL) assay was performed according to the manufacturer’s instructions (*in situ* Cell Detection Kit; Roche, Mannheim, Germany). The methods for TUNEL were performed as previously described (Galvao et al., 2015). Cultured rat RGCs were fixed in 4% (w/v) paraformaldehyde (PFA) at 4°C and were blocked with 3% BSA and 0.1% Triton X-100 for 1 h at RT. The nuclei were counter-stained with DAPI. The preparations were mounted using Glycergel mounting medium (Dako, Denmark) and were visualized using a confocal microscope (Leica).

### Statistical Analysis

All data are expressed as the means ± SEMs and were analyzed using SigmaStat software. All data were tested by the Shapiro-Wilk test and only variables that were normally distributed; the data were then analyzed of variance, followed by two-tailed unpaired Student’s test. Values of *p* < 0.05 were considered the threshold for statistical significance.

## Results

### High Pressure Induces mtDNA Damage and Mutations in Cultured RGCs

**Figure 1:**
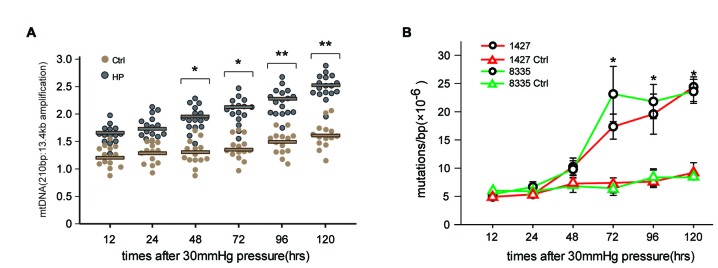
**Elevated hydrostatic pressure (HP; 30 mm Hg) led to mitochondrial DNA (mtDNA) damage and mutations in primarily cultured cells. (A)** mtDNA damage in retinal ganglion cells (RGCs) was determined by long-extension (LX)-PCR, based on the ratio of short to long amplicons at different times after the onset of exposure to increased HP (*n* = 4–7/time point/group). **(B)** After exposure to increased HP, the random mutation capture assay determined that the point mutation frequency in mtDNA increased at two independent sites. *P < 0.05, **P < 0.01. Values are the means ± SEMs. HP, increased hydrostatic pressure; ctrl, control with normal pressure.

To determine whether high pressure itself directly induced mtDNA damage and mutations previously observed in RGCs in glaucomatous rat models, we conducted an *in vitro* experiment to investigate the effects of HP on the mitochondrial genome using cultured RGCs. The exposure of RGCs to high pressure (HP) induced mtDNA damage (indicated by the ratio of the 210 bp and 13.4 kb mtDNA band) as early as 48 h (*p* < 0.05) after onset. The damage continued to increase and was significantly increased (>40%) at 120 h (Figure 1A). Next, we examined the frequency of mtDNA mutations in the RGCs by random mutation capture assay. An increase in mtDNA mutations (1427 and 8335) was observed when the duration of HP was extended beyond 48 h, reaching levels 4.8 ± 0.3-fold (*p* < 0.01), compared with the control values at 120 h (Figure 1B). Furthermore, we found no significant increase in ROS throughout the duration of the experiment (data not shown).

### mtDNA Repair and Replication Compromise After HP

**Figure 2:**
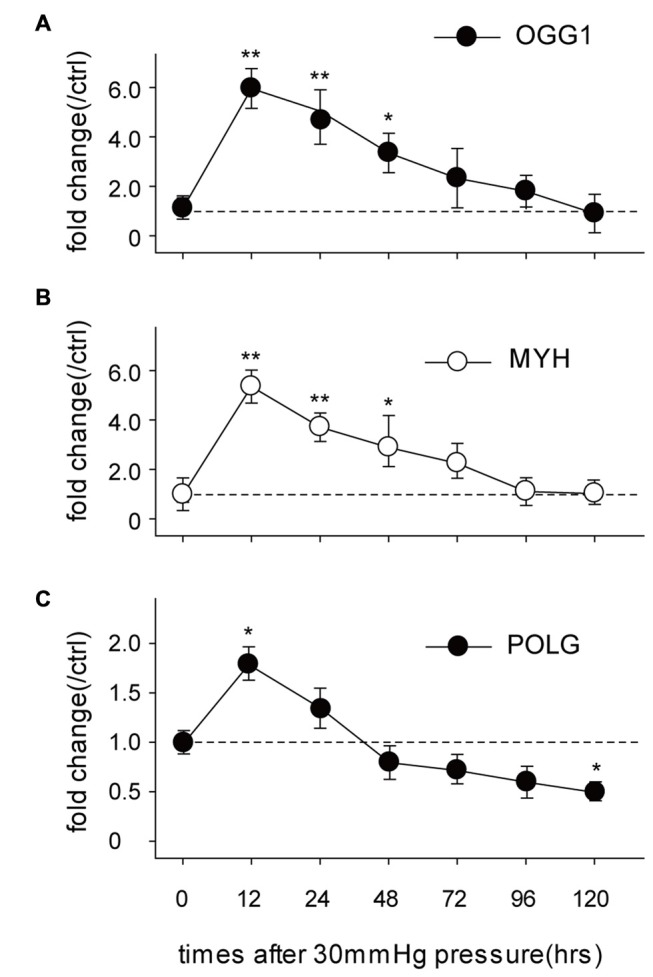
**Real-time PCR analysis of the mRNA levels of the mtDNA repair/replication enzymes OGG1 (A), MYH (B) and polymerase gamma (POLG; C) in the cultured RGCs under an increased HP.** The data are expressed as normalized ratios (Ctrl = 1). Each sample was run in triplicate. (*n* = 5–7/time point/group). *P < 0.05, **P < 0.01. Values are the means ± SEMs. ctrl, control with normal pressure.

**Figure 3:**
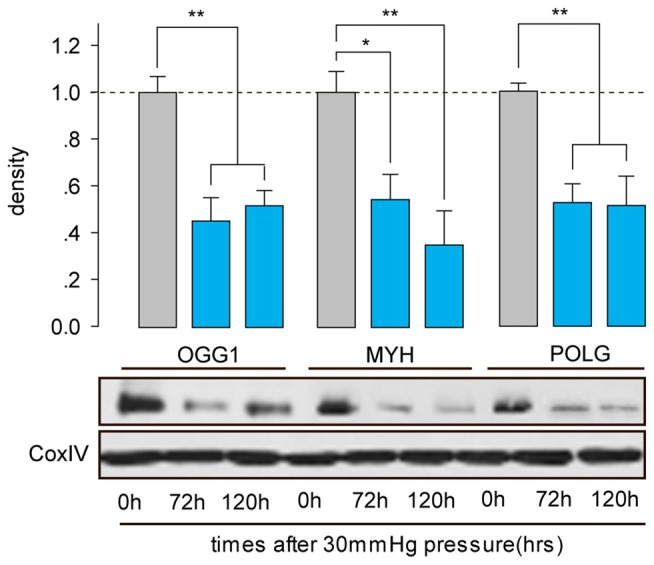
**Western blot analysis showed that protein expressions of OGG1, MYH and POLG in the mitochondria of cultured RGCs decreased after exposure to increased HP.** Cox IV was used as a control to ensure equal protein loading. Band density is expressed in normalized ratios (Ctrl = 1; *n* = 7/time point/group). *P < 0.05, **P < 0.01. Values are the means ± SEMs. HP, increased hydrostatic pressure; ctrl, control with normal pressure.

OGG1, MYH and POLG are important mtDNA repair/replication enzymes. At 12 h of HP exposure, we found that the expression of OGG1, MYH and POLG mRNA in the RGCs were increased 5.02 ± 0.6-fold (*p* < 0.01), 4.3 ± 0.2-fold (*p* < 0.05), and 0.8 ± 0.09-fold (*p* < 0.05), respectively. Subsequently, OGG1 and MYH mRNA levels declined to the levels observed in RGCs incubated under normal pressure (Figures 2A,B), whereas POLG decreased by approximately 1.9 ± 0.4-fold (Figure 2C), compared with control RGCs. In contrast, the mitochondrial accumulation of OGG1, MYH and POLG proteins was significantly decreased at 72 h of HP exposure and remained lower after 120 h of high-pressure insult (Figure 3).

### Mitochondrial Dysfunction After mtDNA Damage

**Figure 4:**
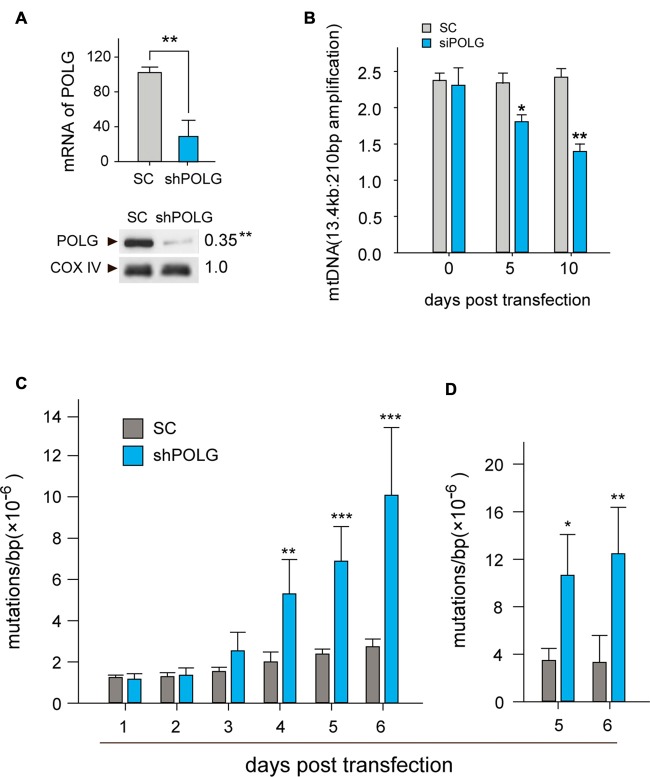
**Transfection of lenti-shPOLG in cultured RGCs led to mtDNA damage and mutations. (A)** POLG mRNA levels were measured by qPCR, and mitochondrial protein levels were quantified by western blot using Cox IV as a loading control at 10 days post-transfection. **(B)** mtDNA damage increased at 10 days after transfection, indicated by the ratios of long (13.4 kb) and short (210 bp) fragments using LX-PCR. Values are presented as the means ± SEMs from three or four independent experiments. **(C,D)** The point mutation frequency in mtDNA increased after transfection was determined by the random mutation capture assay at two independent sites. Values are the means ± SEM. *P < 0.05, **P < 0.01, ***P < 0.001, compared with control RGCs transfected with lenti-scrambled small hairpin RNA (shRNA). ShPOLG, lenti-shPOLG-transfected RGCs; SC, lenti-scrambled shRNA-transfected RGCs.

**Figure 5:**
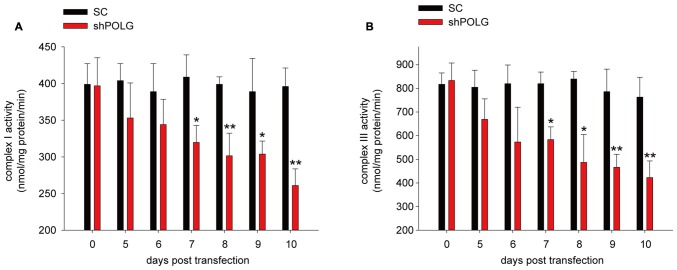
**Mitochondrial complex I (A) and III (B) activity decreased in RGCs transfected with shPOLG, compared to the RGCs with SC shRNA.** Values are the means ± SEMs. *P < 0.05, **P < 0.01, compared with control RGCs transfected with lenti-scrambled shRNA.

**Figure 6:**
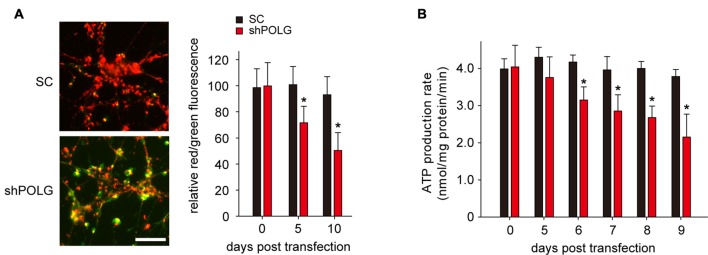
**Transfection of lenti-shPOLG into cultured RGCs led to mitochondrial dysfunction. (A)** Representative images of JC-1 staining of astrocytes at 10 days (left panel). Quantitative analysis of mitochondrial membrane depolarization by flow cytometry after JC-1 staining (right panel). Values are expressed as percentages, with the red/green fluorescence ratio values of lenti-SC shRNA-transfected RGCs set at 100%. Scale bars, 50 μm. **(B)** Mitochondrial ATP production rates in RGCs were analyzed at different time points after transfection with lenti-shPOLG or SC shRNA, using a luciferase-based assay. All of the values in these figures are presented as the means ± SEMs from three or four independent experiments. *P < 0.05, compared with control RGCs transfected with lenti-scrambled shRNA. Sh, lenti-shPOLG-transfected RGCs; SC, lenti-scrambled shRNA-transfected RGCs.

To further determine whether increased mtDNA alterations could lead to mitochondrial dysfunction, we used a lentivirus to transfect POLG-small hairpin RNA (shRNA) into RGCs to block repair of mtDNA alterations, as previously reported (Tewari et al., 2012); subsequently, verification was undertaken by real-time PCR and western blotting. The results showed that the mRNA and protein expression of POLG was significantly decreased 10 days after transfection (*p* < 0.01, Figure 4A). In the lenti-shPOLG-transfected RGCs, mtDNA damage increased, as shown by the decrease in the relative amplification of long (13.4 kb) fragments of mtDNA, compared with short (210 bp) fragments of mtDNA (Figure 4B). The mutation frequency of mtDNA at both 1427 and 8335 sites was also significantly increased (*p* < 0.01) at 4 days after transfection (Figures 4C,D). Then, we examined the changes in mitochondrial function. The activities of complex I and complex III were decreased by 32.6 ± 2.3% (*p* < 0.05) and 46.8 ± 2.8% (*p* < 0.01), respectively, at 10 days after transfection with shPOLG (Figures 5A,B). To further characterize the mitochondrial dysfunction, we used the JC-1 fluorescent probe to measure Δψm, as previously published (Almeida and Bolaños, 2001). The ratio of the fluorescence of the aggregate and monomer forms of JC-1 reflected a decrease in Δψm in shPOLG-treated astrocytes (Figure 6A). We performed the kinetic measurement of ATP production. As expected, the MAPR in the cells transfected with shPOLG decreased in a time-dependent manner, with levels amounting to 57.5 ± 3.7% of those in the SC-transfected cells at 9 days (*p* < 0.05, Figure 6B).

### RGC Apoptosis After mtDNA Damage

**Figure 7:**
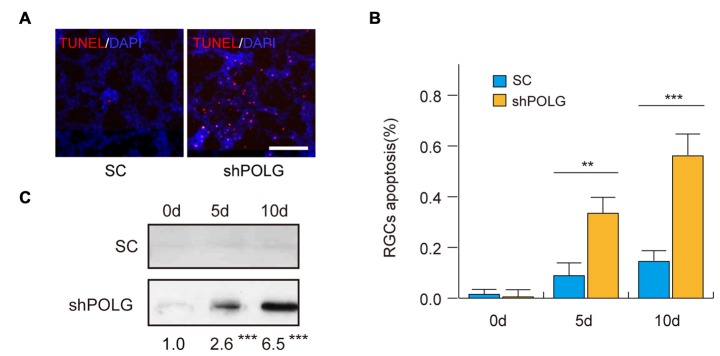
**RGC apoptosis increased after transfection of lenti-shPOLG. (A)** Terminal deoxynucleotidyl transferase dUTP nick end-labeling (TUNEL) assay for detecting apoptotic cell death. Representative microscopic images showing TUNEL-positive cells in the RGCs treated with lenti-shPOLG or with lenti-scrambled shRNA. Red, TUNEL; blue, DAPI. Scale bars, 50 μm. **(B)** Quantitative analysis of RGC apoptosis determined by TUNEL assay. The data are expressed as survival cell counts. **(C)** The active form of caspase-3 increased as determined by western blot using an antibody to cleaved caspase-3. Values are presented as the means ± SEMs. **P < 0.01, ***P < 0.001, compared with control RGCs transfected with lenti-scrambled shRNA. Sh, lenti-shPOLG-transfected RGCs; SC, lenti-scrambled shRNA-transfected RGCs.

Next, we assessed the effects of mtDNA damage and mutation on RGC apoptosis. Thirty-six percentage (*p* < 0.01) and 57% (*p* < 0.001) of RGCs were positive for TUNEL staining 5 and 10 days after transfected with POLG-shRNA, respectively. Indicating the occurrence of cell apoptosis, while no obvious apoptosis was observed in the control group (Figures 7A,B). Meanwhile, western blotting analysis showed that the amount of cleaved caspase-3 was increased 2.6-fold and 6.5-fold 5 and 10 days after transfected with POLG-shRNA, respectively, compared to the controls (*p* < 0.001, Figure 7C).

## Discussion

The present investigation demonstrated the direct relationships among high pressure, mtDNA alterations, mitochondrial dysfunction and RGC apoptosis. Previously, we reported that mtDNA damage and mutations occurred in RGCs in glaucoma and contributed to progressive RGC loss (Wu et al., 2015). Due to the complex pathogenesis of glaucoma, the insult that initiates mtDNA alterations has been unclear. It has been suggested that ROS can result in mtDNA damage (Santos et al., 2011). In fact, it is generally accepted that retinal oxidative stress occurs secondary to IOP elevation (Tezel et al., 2005; Liu et al., 2007). Whether mechanical pressure caused by IOP elevation can directly lead to mtDNA alterations has not yet been investigated. The present study provided evidence that the high pressure could directly cause mtDNA damage and mutations in primarily cultured RGCs, and the mtDNA alterations increased RGC apoptosis. These findings could help to more deeply understand the complex mechanisms of RGC death in glaucoma.

It is well accepted that mtDNA is more vulnerable than nuclear DNA (nDNA) to external stress. In addition, the compromise of the mtDNA repair system can also result in mtDNA alterations (Francisconi et al., 2006; Ciccone et al., 2013; Venesio et al., 2013; Sepe et al., 2016). OGG1, MYH and POLG are important mtDNA repair/replication enzymes. In this study, we found that high pressure induced significant mtDNA alterations as soon as 48 h after onset, and mtDNA alterations occurred before the decreases in OGG1, MYH and POLG expressions after exposure to high pressure, suggesting that mtDNA alterations were apparently initiated by high pressure rather than by impaired mtDNA repair enzymes. Furthermore, the lack of a significant increase in ROS during the experiment diminished the possibility that ROS caused the mtDNA damage or mutations under HP conditions.

This study found that high pressure caused POLG, other than OGG1 and MYH, obviously decreased in the cultured RGCs. This result is consistent with the previous report in the experimental rat glaucoma (Wu et al., 2015). POLG is an important DNA polymerase functioned in mitochondria, which is responsible for mtDNA replication and base excision repair (BER). In the BER, POLG acts as a rate-determining enzyme and its decrease or inactivation may increase the accumulation of deleterious intermediate products, such as DNA single strand breakings (Bohr and Anson, 1999; Ledoux and Wilson, 2001). POLG plays a vital role in maintaining the mtDNA and mitochondrial function (Van Goethem et al., 2001; Di Fonzo et al., 2003). Mutations or knockdown of the POLG gene can cause mtDNA damages, mitochondrial dysfunction and diseases (Van Goethem et al., 2001; Kujoth et al., 2005). It has been reported that POLG1-siRNA could result in mtDNA mutations in retinal endothelial cells (Chen et al., 2002; Graziewicz et al., 2006; Liu and Demple, 2010) and dopaminergic neurons (Dai et al., 2014). Consistent with this finding, in this study, we established an RGC model harboring mtDNA damage and mutations through POLG-shRNA transfection.

ATP and Δψm are important indicators of mitochondrial function. Impaired mitochondrial ATP synthesis and low Δψm are exhibited in cells with mutations in genes encoding the respiratory chain complexes and tRNAs (Schon et al., 2012; Szczepanowska et al., 2012). This study showed the Δψm and ATP production decreased in shPOLG-treated RGCs, suggesting mtDNA alterations were responsible for mitochondrial dysfunction in RGCs. ATP is essential for maintaining Δψm (Xu et al., 2001; Krayl et al., 2007). Recently, Sommer et al. (2016) reported that low Δψm could depress ATP synthesis and transportation. Consistent with this, our data found that Δψm declining followed by impairment of mitochondrial ATP synthesis in shPOLG-treated cells, implying Δψm is a sensitive indicator for mitochondrial function. Mitochondrial dysfunction leads to a reduction of energy production and induction of apoptosis (Osborne, 2010; Lascaratos et al., 2012). Here we indicated that mitochondrial dysfunction resulted from mtDNA mutations significantly increased RGC apoptosis. Moreover, cleaved caspase-3 increased in the shPOLG-transfected RGCs suggested that mitochondria-dependent RGC apoptosis was induced by mtDNA alterations and subsequent mitochondrial dysfunction.

Taken together, the present study further revealed a novel mechanism of RGC death in glaucoma: an elevated IOP could directly cause RGC mtDNA alterations, followed by mitochondrial dysfunction and cell apoptosis.

## Author Contributions

J-HW and X-HS: designed this work, revised it critically and approved the version to be published. S-HZ and F-JG: drafted the manuscript and took part in a majority of the work. J-YC and P-X: took part in some of the experimental studies, for example, western bloting and PCR analysis. Z-MS: analysis of experimental results.

## Conflict of Interest Statement

The authors declare that the research was conducted in the absence of any commercial or financial relationships that could be construed as a potential conflict of interest.
